# Salmagundi

**Published:** 1884-06

**Authors:** 


### SALMAGUNDI.

EDITED BY E. G. BETTY, D.D.S.

TARNISHED nickel can be polished with chalk or rouge mixed with tallow.

There are published in the United States more than twenty dental periodicals.

LISTERINE is becoming more popular every day with the dental profession, because, to use a trite phrase, "it supplies a long-felt want."

PLATINUM can be drawn into wire 0.00009 of an inch in diameter, a fineness absolutely invisible to the naked eye, though capable of sustaining a weight of four grains.

---

DR. SAMUEL WARDLE, of Cincinnati, is the only dentist in that city who knows how to carve teeth for himself. "Pity 'tis, 'tis true," for if there were more like him we would see fewer of the store teeth that grin their ghastly grins at every turn one makes.

---

Massachusetts, containing as it does the "hub of the universe" with all that implies, to say nothing of Transcen-*dental* Philosophy, is without "Concord" on the subject of qualification for practicing dentistry, inasmuch as it has no law whatever regulating it, though the good "Shephard" may in time gather the lambs of the flock into one fold.

---

CAULK'S DENTAL ANNUAL, second edition for 1883–84, is at hand, with many additions to satistical tables, revised and corrected up to date of going to press. We wish the enterprise all the success it deserves, for this is the age of statistics and every effort at collecting and classifying them adds another stone to the foundation upon which we base our practice.

---

New Jersey and Missouri are the only states in the Union that require *graduation* as the sole and absolute condition upon which dentists are permitted to practice within their boundaries. If all the states boasting of proud colleges and universities were this day to compel graduation at a reputable institution, dentistry as practiced by the bulk of those engaged in it would cease to be regarded by the public as a genteel trade.

---

"IT is related of Dr. Gross, the distinguished surgeon who has just died, that he adhered to the use of chloroform, and out of over five thousand cases in which it was administered under his careful supervision, not one accident occurred." In this connection we may mention that Dr. Watt holds to the practice of giving one anæsthetic alone; that is, either chloroform, ether, etc.; not mixing them as is frequently done, for the reason that the symptoms can be read better and with distinctness, and of course the anæsthesia controlled with more certainty, thus lessening the danger of accident.

---

A New York man went home late, and being under suspicion, his wife went through his pockets and found a set of false teeth, which were identified as the property of her rival. There was a bedroom scene; and, on the following day, a court-room scene, but furthermore the record is not lucid. However enough is told to serve as a warning against married men carrying home sets of teeth as souvenirs of love.—*Daily Paper.*

---

A cablegram to a daily paper, dated May 19, says that M. Pasteur has discovered a method to prevent hydrophobia. The virus of a rabid dog taken from the brain is used to inoculate a monkey, and virus from this one introduced into another, and so on through six or seven. By this time the poison has become so weakened that a dog inoculated with it does not become affected with the disease, even though subsequently exposed to original virus. On the other hand, the diluted virus from the last monkey is made to regain its original potency by passing it successively through six or seven rabbits.

---

The National Museum at Naples, Italy, contains a collection of ancient tools found during the excavation in the ruins of Pompeii. Among them are some of very fine structure and workmanship, and judging from their form it is almost certain that they are the instruments once used by the dentist of near twenty centuries ago. This seems to be corroborated (in fact almost proved beyond doubt) by finding upon fragments of the "Twelve Tablets" a penal codex dating from the earliest Roman days, forbidding persons to extract the teeth from the mouths of the dead for the sake of the gold contained in them: "*Cin dentes·auro vincti escunt* (*sunt*)." The above is from a translation in a German periodical, kindly made for us.

---

In several particulars concerning dental matters, Ohio ranks second and fifth in the list of States. Thus she is *second* in the founding of a dental college, the passage of laws regulating practice, and the establishment of a professional periodical, and *fifth* in the number of her dentists; the number of members belonging to the State Society (not counting New York, number unknown), and is a tie for that place, with Connecticut, Georgia, Indiana, and New Jersey, for the number of dealers and manufacturers. She is also first in the number of great dentists she has developed, and the organization of the oldest dental society in the world, whose annual meetings have for many years been held in her principal city "on the banks of the beautiful river." So, averaging the thing up, her people have a tolerable idea of what good dentistry is and ought to be.

A reformed drinker writing in an exchange says: I was one of those unfortunates given to strong drink. When I left it off I felt a horrid want of something I must have or go distracted. I could neither eat, work, nor sleep. Explaining my affliction to a man of much education and experience, he advised me to make a decoction of ground quassia, a half ounce steeped in a pint of vinegar, and to put about a teaspoonful of it in a little water, and to drink it down every time the liquor thirst came on me violently. I found it satisfied the cravings, and it suffused a feeling of stimulus and strength. I continued this cure, and persevered till the thirst was conquered. For two years I have not tasted liquor, and I have no desire for it. Lately, to try my strength, I have handled and smelt whisky, but I have no temptation to take it. I give this for the consideration of the unfortunate, several of whom I know have recovered by means which I no longer require."

